# Supplementary material for: Identification of Three Viruses Infecting Mulberry Varieties
Source: Viruses. 2022 Nov 19;14(11):2564. doi: 10.3390/v14112564 (PMC9696721; doi:10.3390/v14112564)
Supplement: Supplementary file 1 [file viruses-14-02564-s001.zip › viruses-2019611-supplementary.pdf]

Table S1. The primers of RACE

Name	Primer sequences[1]	
10×UPM	CTAATACGACTCACTATAGGGCAAGCAGTGGTATCAACGCAGAG T	
CLBV2-5UTR-R	GATTACGCCAAGCTTCATAACTACGGAATCGATC	GSP
CLBV2-5UTR-R1	CCCTTCTCTCCACCTCAACAACCAGCG	GSP
CLBV2-3UTR-F	GATTACGCCAAGCTTGGATCAAGGAGTACATCAGACA	GSP
CLBV2-3UTR-F1	GGCGGGTGCTGAGGCGGAAGAT	GSP
M13-F	CAGGGTTTTCCCAGTCACG	
M13-R	GAGCGGATAACAATTTCACAC	
CLBV2-RdRp-det-F	GAAGATAGTGTCAATGCATTTG	
CLBV2-RdRp-det-R	CCAGCCACAAAACATCGGGCTC	
MaTLV-RdRp-det-F	AGCGACAAAAATTTTTCTTCATCC	
MaTLV-RdRp-det-R	CGCGCGAAGGAAAAAACCTACG	
MaNLV-RdRp-det-F	GTATCACCAAGGTAATTCTTACC	
MaNLV-RdRp-det-R	ATCACCGCTCACCAAGAGAACG	

[1] the 15 bp overlaps with the pUC19 vector.

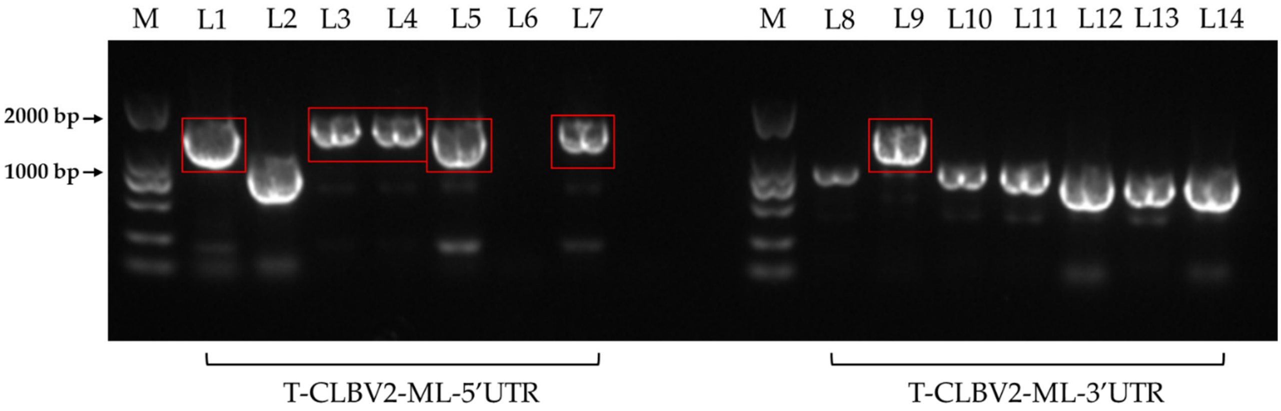

Figure S1. The detection of positive clones; M was Marker, Lane 1-7 were the positive clones of 5' terminal sequences, Lane 8-14 were the positive clones of 3' terminal sequences; The stripes in the red boxes maybe the destination stripe.

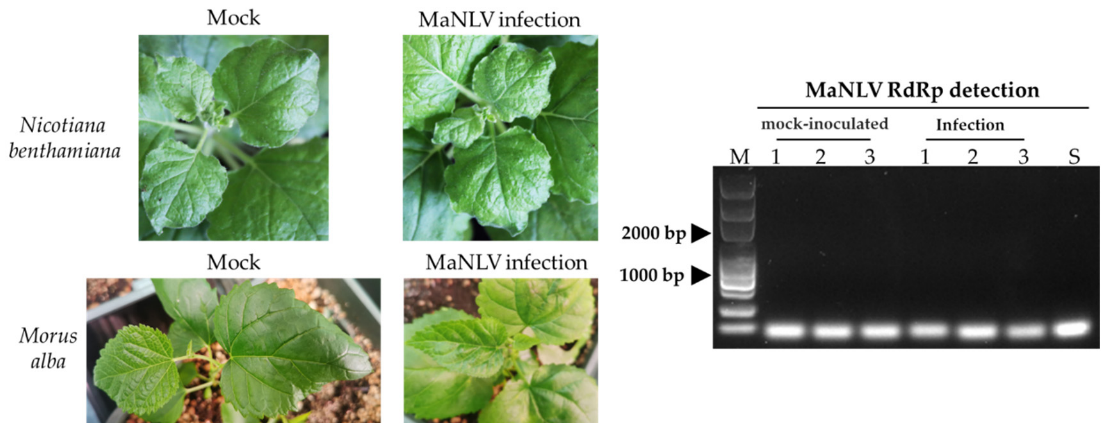

Figure S2. Symptoms on leaves of *Nicotiana benthamiana* and *Morus alba* infected by MaNLV, and virus detection. Lane 1 and 2, *N. benthamiana*; Lane 3, *Moru alba*; m1-3, mock-inoculated leaves; i1-3, the infection of leaves; S, sample cDNA.
